# The Pathology of Cerebral Hemorrhage

**Published:** 1871-04

**Authors:** 


### THE PATHOLOGY OF CEREBRAL HEMORRHAGE.

Charcot and Bouchard, examining the causes to which cerebral hemorrhages are usually referred, find that they may be arranged in three groups: (1) Diminution of the consistence of cerebral tissue to such a degree that it does not furnish sufficient support to the vessels; (2) Increased tension of the blood, depending on hypertrophy of the left venticle, atrophy of the kidneys, etc.; (3) Dimished resistance of the vessel in consequence of change in the walls (fatty degeneration or atheromatous incrustation.)

Some of these (as hemorrhagic *ramollisement*, for example,) are doubtful; others appear to be only accessory. This the authors find, with regard to arterial atheroma, that it is not present in twenty-two per cent. of cases, and that it is only present in a marked degree in twenty-five per cent. Again, hypertrophy of the heart was not present in forty per cent. of cases. These statistics are founded on examination of sixty-nine cases. The only pathological condition which MM. Charcot and Bouchard have constantly met is an aneurismal state of a certain number of the small intra-cerebral vessels. From the small size of these aneurisms the authors give them the name of miliary aneurisms. They are visible to the naked eye. They appear as small globular grains, varying in diameter from two-tenths of a millimetre to a millimetre, and sometimes a little larger. If they contain liquid blood, their color is red or velvit; if, on the other hand, the blood is concreted or has undergone transformation, the aneurism is red-brown, ochry, or even blackish. The color is so influenced by the variable thickness of the wall. The optic thalami, the corpora striata, the convolutions, the pons, the cerebellum, the centrum ovale, the middle peduncles of the cerebellum, the cerebral peduncles, the medulla oblongata, are the parts in order of frequency where these aneurismal dilatations have been most frequently met by the authors.

This distribution, they remark, agrees with that of cerebral hemorrhages. Seen under the microscope with a low power, the vessels present a fusiform or sacciform dilatation. With a higher power it is seen that the wall of the aneurism is continuous with the tunics of the vessel which is its seat, but the three tunics are not distinct, they are fused, and the thickness of the aneurismal wall is less than that of the three normal coats of the vessel, a fact which accounts for their proneness to rupture. Careful observation of the aneurismal vessel reveals alteration of structure (*arterite sclereuse*). This change proceeds from without inwards, for the most considerate changes have their seat in the outer parts of the vessel, and the atrophy of the muscular coat depends on change in the adventitious tunic—whence the name periarteritis which they propose for it.—*Half-Yearly Abstract.*

